# PRDM14 promotes RAG-dependent *Notch1* driver mutations in mouse T-ALL

**DOI:** 10.1242/bio.017699

**Published:** 2016-04-22

**Authors:** Brandi L. Carofino, Bernard Ayanga, Lauren J. Tracey, Travis Brooke-Bisschop, Monica J. Justice

**Affiliations:** 1 Interdepartmental Program in Translational Biology and Molecular Medicine, Baylor College of Medicine, Houston, TX, 77030 USA; 2 Department of Molecular and Human Genetics, Baylor College of Medicine, Houston, TX, 77030 USA; 3 Department of Molecular Genetics, University of Toronto, Toronto, Ontario, M5S 1A8 Canada; 4 Genetics and Genome Biology, The Hospital for Sick Children, The Peter Gilgan Centre for Research and Learning, Toronto, Ontario, M5G 0A4 Canada

**Keywords:** PRDM14, NOTCH1, Driver mutation, RAG recombination, T-cell acute lymphoblastic leukemia

## Abstract

PRDM14 is an epigenetic regulator known for maintaining embryonic stem cell identity and resetting potency in primordial germ cells. However, hematopoietic expression of *Prdm14* at supraphysiological levels results in fully penetrant and rapid-onset T-cell acute lymphoblastic leukemia (T-ALL) in the mouse. Here, we show that PRDM14-induced T-ALLs are driven by NOTCH1, a frequently mutated driver of human T-ALL. *Notch1* is activated in this murine model via RAG-dependent promoter deletions and subsequent production of truncated, ligand-independent protein from downstream regions of the *Notch1* locus. These T-ALLs also have focal changes in H3K4me3 deposition at the *Notch1* locus and global increases in both H3K4me1 and H3K4me3. Using a PRDM14-FLAG mouse model, we show that PRDM14 binds within an intron of *Notch1* prior to leukemia development. Our data support the idea that PRDM14 binding promotes a chromatin state that allows access of the RAG recombinase complex to cryptic RAG signal sequences embedded at the *Notch1* locus. Indeed, breeding into a RAG recombination-deficient background abrogates T-ALL development and prevents *Notch1* deletions, while allowing for transient hematopoietic stem cell (HSC)-like pre-leukemia cell expansion. Together, our data suggest that PRDM14 expands a progenitor cell population while promoting a permissive epigenetic state for the creation of driver mutations (here, in *Notch1*), enabling cancer development through the misappropriation of endogenous cellular DNA recombination machinery.

## Abstract (precis)

**Summary:** PRDM14 promotes an epigenetic state that facilitates RAG-dependent *Notch1* driver mutations, coupling progenitor cell expansion with genomic instability to produce T-ALL with shorter latency than other NOTCH1-driven mouse models.

## INTRODUCTION

Factors that control normal growth and differentiation are often the main drivers of neoplasia. Members of the PR domain-containing (PRDM) protein family are frequently deregulated in hematological malignancies (Fog et al., 2012). The PR domain shares 20-30% amino acid sequence identity with the SET domain, the catalytic component of most histone methyltransferases (HMTases) (Herz et al., 2013). Despite this homology, HMTase activity has been reported for only a small number of PRDM family members, suggesting that other PRDMs involved in epigenetic gene regulation may do so via association with catalytically active protein complexes (Fog et al., 2012).

PRDM14 has been implicated in a large number of human solid tumors (Moelans et al., 2010; Nishikawa et al., 2007; Ruark et al., 2013; Steenbergen et al., 2013; Terashima et al., 2014; Zhang et al., 2013) and acute lymphoid leukemias (Dettman et al., 2011). During normal development, however, *Prdm14* expression is restricted to pluripotent cell types, including primordial germ cells (PGCs) (Yamaji et al., 2008), 2- to 8-cell stage embryos (Burton et al., 2013), the murine inner cell mass (Burton et al., 2013), and both murine (Ma et al., 2011) and human (Tsuneyoshi et al., 2008) embryonic stem cells (ESCs). In PGC specification, PRDM14 supports pluripotency factor expression, reduces DNA methylation, and alters histone methylation (Yamaji et al., 2008). In ESCs, PRDM14 supports the maintenance of naïve pluripotency by global DNA hypomethylation and by interacting with polycomb repressive complex 2 (PRC2) to repress differentiation genes (Chan et al., 2013; Ma et al., 2011; Tsuneyoshi et al., 2008).

We previously described *Prdm14* as a potent leukemia oncogene using a Cre recombinase-inducible ROSA26-*lox*P-STOP-*lox*P*-Prdm14*-IRES-eGFP (R26PR) mouse model (Carofino et al., 2013). R26PR mice develop fully penetrant T-cell acute lymphoblastic leukemia (T-ALL) with exceptionally short latency (<2 months) when the allele is activated using hematopoietic stem cell-expressing Cre transgenic lines, including Mx1-cre and MMTV-cre. Prior to the accumulation of malignant thymocytes, R26PR;cre mice exhibit a dramatic expansion of cells with an immunophenotype similar to long-term hematopoietic stem cells (LT-HSCs), indicating that PRDM14 may drive LT-HSCs from a quiescent to proliferative state. Furthermore, these LT-HSC-like cells preferentially give rise to common lymphoid progenitors (CLPs) and T-cells, with concomitant blocks in myeloid and B-cell differentiation, suggesting that PRDM14 or downstream factors influence hematopoietic lineage-fate decisions.

A candidate cooperating driver of this phenotype is NOTCH1, which is mutated in greater than 50% of human T-ALL cases (Weng et al., 2004). NOTCH1 plays vital roles in HSC self-renewal, lymphoid versus myeloid lineage fate decisions, and T-cell development (Radtke et al., 1999; Stier et al., 2002). Mice transplanted with bone marrow expressing activated *Notch1* alleles develop exclusively T-cell leukemias (Pear et al., 1996). R26PR-derived tumors and tumor cell lines overexpress NOTCH1 and are sensitive to pharmacological inhibition of NOTCH1 protein processing (Carofino et al., 2013).

NOTCH1 is a transmembrane receptor that is activated following ligand binding, which triggers proteolytic cleavage by ADAM-family metalloproteases and subsequently by gamma-secretase. NOTCH1 intracellular domain (NICD) then translocates to the nucleus to activate downstream genes (Fortini, 2009). *NOTCH1* mutations found in both humans and mice result in an abnormal accumulation of NICD and activation of NOTCH1 target genes. The most prevalent human leukemia-associated *NOTCH1* mutations occur in the heterodimerization domain of the NOTCH1 negative regulatory region. These mutations destabilize the interaction of the extracellular and intracellular portions of NOTCH1 and lead to ligand-independent NICD release (Malecki et al., 2006). The second most common class of mutations fall in the C-terminal PEST domain, which marks NICD for ubiquitination and proteasomal degradation, leading to reduced protein turnover (Weng et al., 2003). In mice, the most common mutations, termed ‘type 1’ deletions, involve recombination-activating gene (RAG) complex-mediated 5′ *Notch1* deletions that drive expression from a downstream transcription start site, producing a truncated protein that is constitutively cleaved by gamma-secretase (Ashworth et al., 2010; Tsuji et al., 2004, 2009). These ‘type 1’ deletion breakpoints are flanked by cryptic RAG recombination signal sequences (cRSSs), which bear homology to the canonical RSSs that mediate V(D)J recombination within antigen receptor genes. As a complex, RAG1 endonuclease binds to the RSS, while RAG2 binds to trimethylated histone 3 lysine 4 (H3K4me3). Both RAG2 and H3K4me3 binding, as assessed by chromatin immunoprecipitation (ChIP), is enriched at the 5′ end of *Notch1* in normal thymocytes (Ashworth et al., 2010)*.* RAG1 and RAG2 colocalize at thousands of sites in the lymphocyte genome, but selective pressure has reduced the abundance of cRSSs in close proximity to transcriptionally active loci containing H3K4me3 (Teng et al., 2015). Despite this, illegitimate RAG recombination still accounts for many of the chromosomal translocations commonly observed in leukemias (Haines et al., 2006).

Here, we show that PRDM14 binds to the *Notch1* locus prior to leukemia onset. Regions of the *Notch1* locus become enriched for H3K4me3, which is required for RAG2 binding. These epigenetic changes facilitate RAG-mediated deletion of the canonical *Notch1* promoter and expression of truncated *Notch1* from its intragenic promoter. We also show that PRDM14-induced *Notch1* deletions are RAG-dependent while the pre-leukemic HSC-like cell expansion is RAG independent. Therefore, PRDM14 may induce T-ALL by promoting a pre-malignant expansion of progenitor cells carrying an epigenetic state that predisposes them to accumulate driver mutations. Here, the driver mutation is within a potent T-cell leukemia oncogene, *Notch1*.

## RESULTS

### PRDM14-induced T-ALLs carry *Notch1* 5′ deletions that produce NICD

We previously showed that induction of R26PR in HSCs by Mx1-cre or MMTV-cre produced exclusively pre-T-cell ALLs, which express high levels of *Notch1* mRNA, NICD protein, and NICD target genes (Carofino et al., 2013). Because of experimental limitations resulting from the lack of a ChIP-grade PRDM14 antibody, we generated another inducible mouse line, ROSA26-*lox*P-STOP-*lox*P-3XFLAG-PRDM14-P2A-eGFP (R26FLPR), which is similar to R26PR in construction except for the addition of an N-terminal 3×FLAG-tag to PRDM14 and the use of viral peptide 2A (P2A) instead of an internal ribosome entry site (IRES). R26FLPR mice expressed FLAG-PRDM14 protein of the expected molecular weight (∼65 kDa) and a higher molecular weight band of unknown identity; while it is possible for a FLAG-PRDM14-GFP fusion protein to arise from inefficient P2A cleavage, P2A is the most efficient of the 2A peptides (Kim et al., 2011) and the upper band did not cross-react with an anti-GFP antibody. Most R26FLPR;Mx1-cre mice succumbed to a rapid-onset T-ALL that was phenotypically comparable to the original model, but with variable penetrance depending on the Cre line used (Fig. S1). Furthermore, R26FLPR thymic tumors expressed NICD (Fig. S2A).

**Fig. 1.**
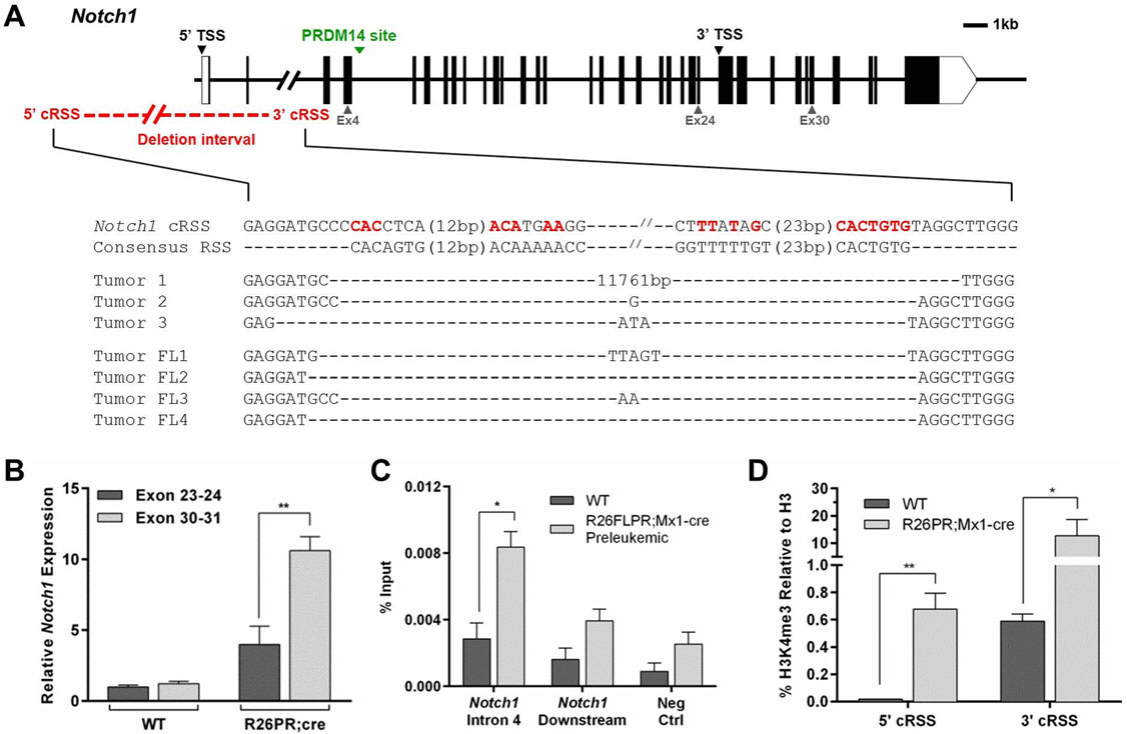
***Notch1* is mutated and bears focal epigenetic changes in PRDM14-expressing cells.** (A) Upper, schematic of the *Notch1* locus. Exons are shown as black boxes. The region deleted in PRDM14-induced tumors, the canonical transcription start site (5′ TSS), the downstream 3′ TSS, and the putative PRDM14 binding site are indicated. Lower, alignment of sequences from PRDM14-induced tumors with the consensus RSS and cryptic RSS (cRSS) found at *Notch1*. Conserved nucleotides are shown in red. (B) Ratiometric qRT-PCR using primers spanning *Notch1* exon 23-24 and *Notch1* exon 30-31 in wild-type (WT) (*n*=5) and R26PR;cre (*n*=7) thymi. Values shown are relative to one wild-type animal. (C) ChIP-qPCR for FLAG-PRDM14 at the putative PRDM14 binding site in *Notch1* intron 4, a non-specific intronic site 2.5 kb downstream of the putative *Notch1* binding site, and a gene desert located on Chr 6 that contains no genes (Neg Ctrl) in unsorted whole bone marrow of wild-type (*n*=3) and R26FLPR;Mx1-cre (*n*=3) mice at 10 days post-pIpC injection. (D) ChIP-qPCR for H3K4me3 at the *Notch1* 5′ and 3′ cRSSs in wild-type (*n*=5) and R26PR;Mx1-cre (*n*=5) thymi. Mean±s.d. shown for all graphs. *P≤0.05, **P≤0.01.

We assayed for *Notch1* mutations in R26PR;Mx1-cre and R26FLPR;Mx1-cre thymic tumors by sequencing the 5′ promoter region. In tumors from both lines, we observed cRSS-flanked promoter deletions at *Notch1* consistent with those previously characterized as ‘type 1’ deletions (Ashworth et al., 2010) (Fig. 1A). A ratiometric qRT-PCR assay was carried out to determine the levels of *Notch1* expressed from the 5′ transcriptional start site (5′ TSS; full length, containing exons 1-34) and the intragenic transcriptional start site (3′ TSS; containing exons 25-34), which is expected to generate a ligand-independent form of NOTCH1 in ‘type 1’ deletions. R26PR tumors showed a significant excess of 3′ transcript, indicative of 3′ TSS usage, while wild-type thymi expressed equal amounts of 5′ and 3′ transcripts (Fig. 1B).

Transcription from the intragenic *Notch1* promoter also requires displacement of IKAROS, which mediates the ability of transcriptional machinery to access this region (Gómez-del Arco et al., 2010; Jeannet et al., 2010). H3K4me3, a mark of active transcription, was enriched at the 3′ TSS within R26PR;Mx1-cre thymic tumors, while less IKAROS was bound (Fig. S3A,B) within tumors. No changes in *Ikaros* expression were detected that could account for the loss of binding (Fig. S2, Fig. S3C). Therefore, *Notch1* was transcribed from the 3′ TSS in PRDM14-induced tumors.

### PRDM14 binds to the *Notch1* locus prior to leukemia onset

PRDM14 has a putative binding site in *Notch1* intron 4, between its canonical transcription start site (5′ TSS) and the intragenic 3′ TSS (Fig. 1A). Therefore, we performed ChIP for FLAG in unsorted whole bone marrow (BM), which contains a mixture of FLAG-PRDM14 expressing and non-expressing cells, of R26FLPR;Mx1-cre mice 10 days after induction of *Prdm14* expression to determine if PRDM14 binds to this region prior to leukemia development. At this time point, FLAG-PRDM14 was expressed, but NICD was not yet overexpressed in all samples (Fig. S2B). We observed a significant enrichment of FLAG at *Notch1* intron 4, but not at downstream or negative control regions (Fig. 1C). Thus, PRDM14 binds to the *Notch1* locus prior to disease onset.

### PRDM14-induced tumors have focal and global epigenetic alterations

**Fig. 2.**
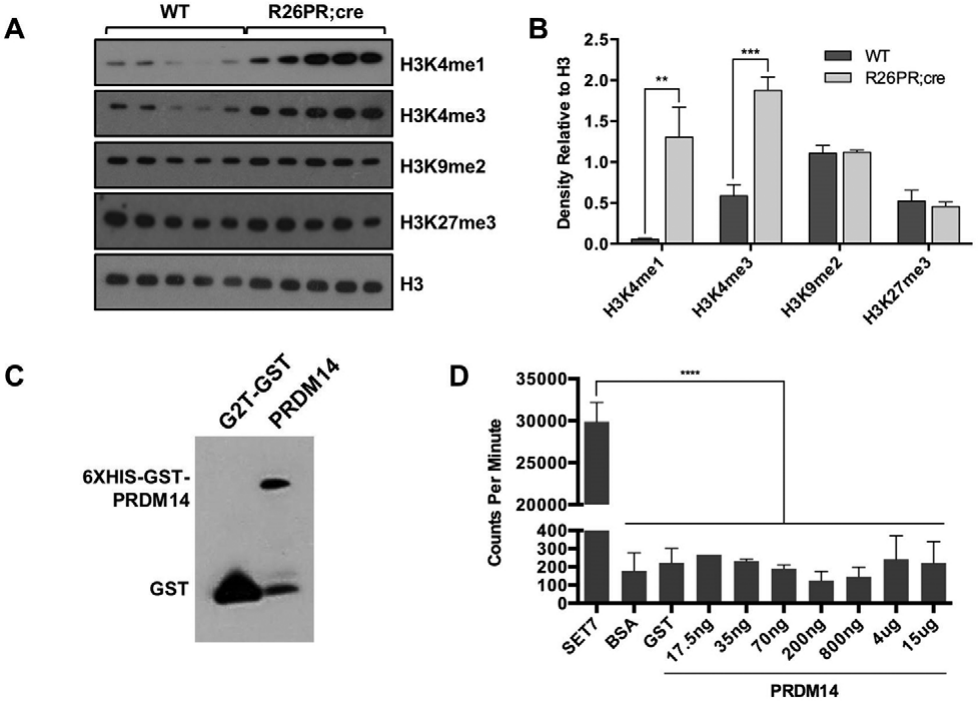
**Global epigenetic changes in PRDM14-induced T-ALLs.** (A) Western blot for global histone modifications using histone extracts prepared from wild-type (WT) thymi (*n*=5) and R26PR;cre thymic tumors (*n*=5). (B) Densitometric quantification of A, with whole H3 used for normalization. (C) Western blot for GST on purified recombinant G2T-GST (control; 26 kDa) and GST-PRDM14 (90 kDa) protein. (D) H3 HMTase activity assay for positive control (SET7), negative control (BSA, GST), and PRDM14 recombinant proteins. Mean±s.d. shown for all graphs. **P≤0.01, ***P≤0.001, ***P≤0.0001.

Because PRDM14 expression is accompanied by changes in chromatin marks in pluripotent cells and RAG2 recruitment requires H3K4me3, we assayed for focal H3K4me3 at the *Notch1* cRSSs and for global histone modifications in PRDM14-induced tumors. Both the 5′ and 3′ *Notch1* cRSSs were significantly enriched for H3K4me3 in tumors but not wild-type thymi (Fig. 1D). We also examined H3K4me1 and H3K4me3 at the putative PRDM14 binding site in *Notch1* intron 4 in pre-leukemic BM, but neither mark was significantly enriched at this early time point in whole bone marrow (data not shown). However, a global increase in both H3K4me1 and H3K4me3, activating chromatin marks, but not H3K9me2 or H3K27me3, repressive chromatin marks, was observed in tumors (Fig. 2A,B).

Despite its repeated association with histone modifications, PRDM14 has never been demonstrated to have HMTase activity. Therefore, we purified recombinant PRDM14 from a eukaryotic expression system (Fig. 2C) to perform an *in vitro* HMTase activity assay. SET7, a known HMTase, but not GST, BSA (negative control proteins), or varying amounts of PRDM14, catalyzed the transfer of a methyl group to recombinant H3 protein (Fig. 2D). Therefore, PRDM14 does not have HMTase activity in this assay.

### RAG recombination is required for *Notch1* deletions and T-ALL development

**Fig. 3.**
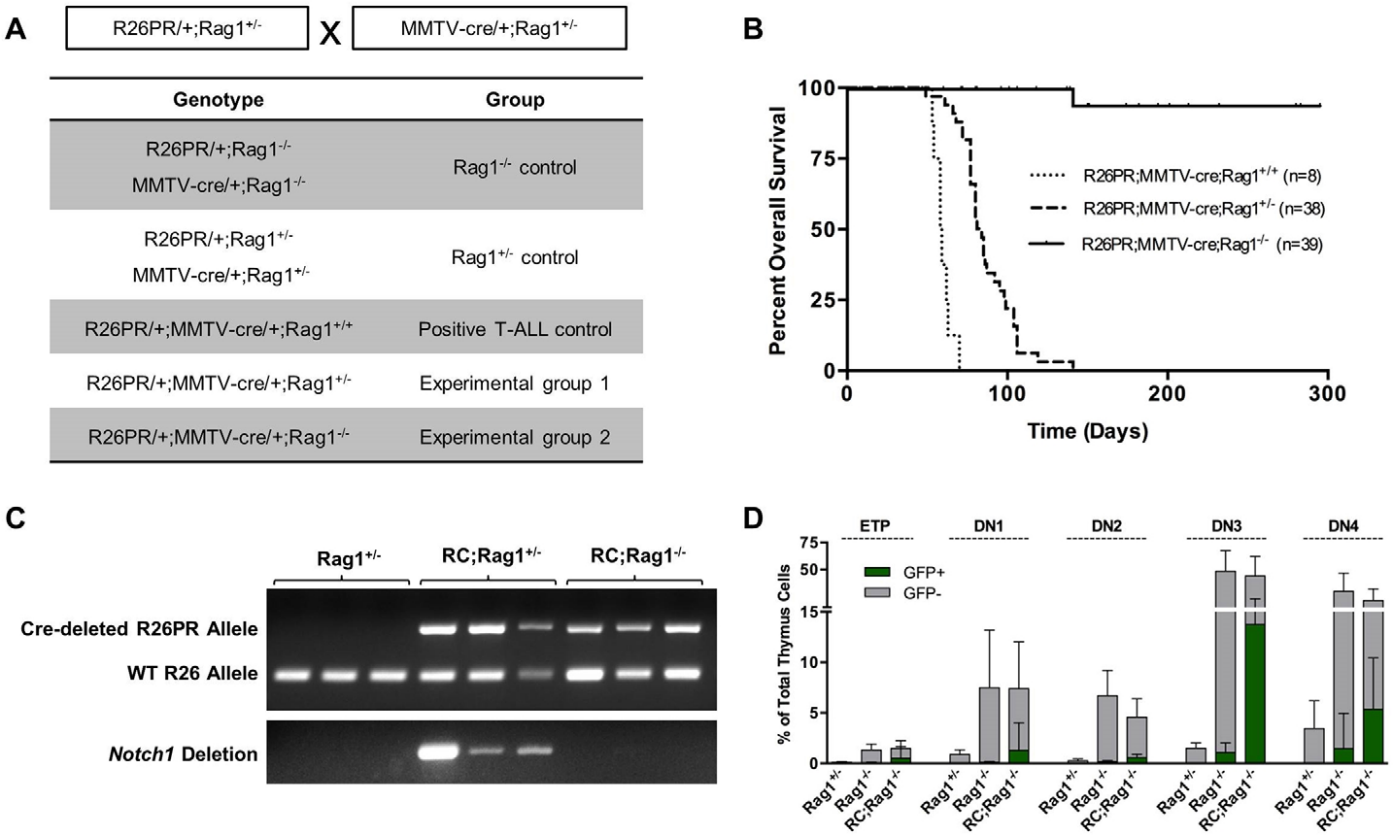
**RAG recombination-impaired R26PR;cre mice do not develop leukemia or carry *Notch1* deletions.** (A) Breeding scheme and genotype grouping of *Rag1* mutant mouse lines. (B) Overall survival of R26PR;MMTV-cre;*Rag1^+/+^* (*n*=8), R26PR;MMTV-cre;*Rag1*^+/−^ (*n*=38), and R26PR;MMTV-cre;*Rag1*^−/−^ (*n*=39) mice. (C) PCR-based verification of R26PR allele status following exposure to Cre recombinase (upper) and PCR-based detection of *Notch1* deletion status in R26PR;MMTV-cre mice with or without *Rag1* (lower). RC, R26PR;MMTV-cre. (D) Flow cytometric analyses of early thymocyte development in the thymi of *Rag1*^+/−^ (*n*=13), *Rag1*^−/−^ (*n*=15), and R26PR;MMTV-cre;*Rag1*^−/−^ (*n*=14) mice. Thymocytes were stained for CD4, CD8, c-kit, CD44, and CD25, then double negative (DN; CD4^−^CD8^−^) cells were separated into stages according the expression of c-kit, CD44, and CD25. ETPs, CD4^−^CD8^−^CD44^+^CD25^−^c-kit^+^; DN1, CD4^−^CD8^−^CD44^+^CD25^−^; DN2, CD4^−^CD8^−^CD44^+^CD25^+^; DN3, CD4^−^CD8^−^CD44^−^CD25^+^; DN4, CD4^−^CD8^−^CD44^−^CD25^−^. GFP-positive and -negative cells are displayed as green and gray bars, respectively, and mean±s.d. is shown for all graphs. RC, R26PR;MMTV-cre.

The presence of the RAG complex may facilitate the accumulation of *Notch1* mutations by promoting recombination at accessible cRSSs in PRDM14-induced T-ALLs. Therefore, we generated mice carrying both R26PR and MMTV-cre, which were also null or heterozygous for the *Rag1* gene (Fig. 3A). *Rag1* null mice produce no mature B- or T-cells because they cannot form functional antigen receptors (Mombaerts et al., 1992).

Each genotype was aged and assessed for phenotype (Fig. 3B). Negative control genotypes carrying only R26PR or MMTV-cre along with *Rag1*^−/−^ or *Rag1*^+/−^ did not develop tumors (data not shown). R26PR;MMTV-cre;*Rag1*^+/+^ mice developed T-ALL with a median survival of 58.5 days of age, which was similar to the survival observed in our previous study (Carofino et al., 2013). The R26PR;MMTV-cre;*Rag1*^+/−^ mice had a slightly longer latency of disease onset and median survival of 84 days. However, only 1/39 animals with the R26PR;MMTV-cre;*Rag1*^−/−^ genotype developed T-ALL. These animals did not develop leukemia even when aged to nearly 300 days, although some animals developed infections. Deletion of the floxed-STOP cassette was confirmed with PCR to verify that the R26PR allele status was identical in all mice irrespective of tumor status. Furthermore, *Notch1* deletions were only detected in R26PR;MMTV-cre mice with intact RAG activity (Fig. 3C), suggesting that RAG deficiency prevents the accumulation of *Notch1* driver mutations in R26PR;MMTV-cre mice.

In a RAG-deficient background, thymocyte development is blocked because of the inability to produce functional T-cell receptors (TCRs). The first thymic emigrant progenitor cells (early thymic progenitors, ETPs) progress through four double negative (DN) stages. RAG-mediated recombination of TCR loci begins at DN2, and DN3 cells undergo β-selection for a successfully rearranged TCRβ gene (Miosge and Goodnow, 2005). Therefore, *Rag1*-deficient thymocytes are expected to arrest at DN3. Indeed, both *Rag1*^−/−^ and R26PR;MMTV-cre;*Rag1*^−/−^ thymocytes accumulate at the DN3 stage (mean 48.5% and 44%, Fig. 3D), suggesting that *Prdm14* expression is not sufficient to drive *Rag1*^−/−^ thymocytes past the β-selection checkpoint.

### LT-HSCs and CLPs are expanded in R26PR;MMTV-cre;*Rag1^−/−^* mice

**Fig. 4.**
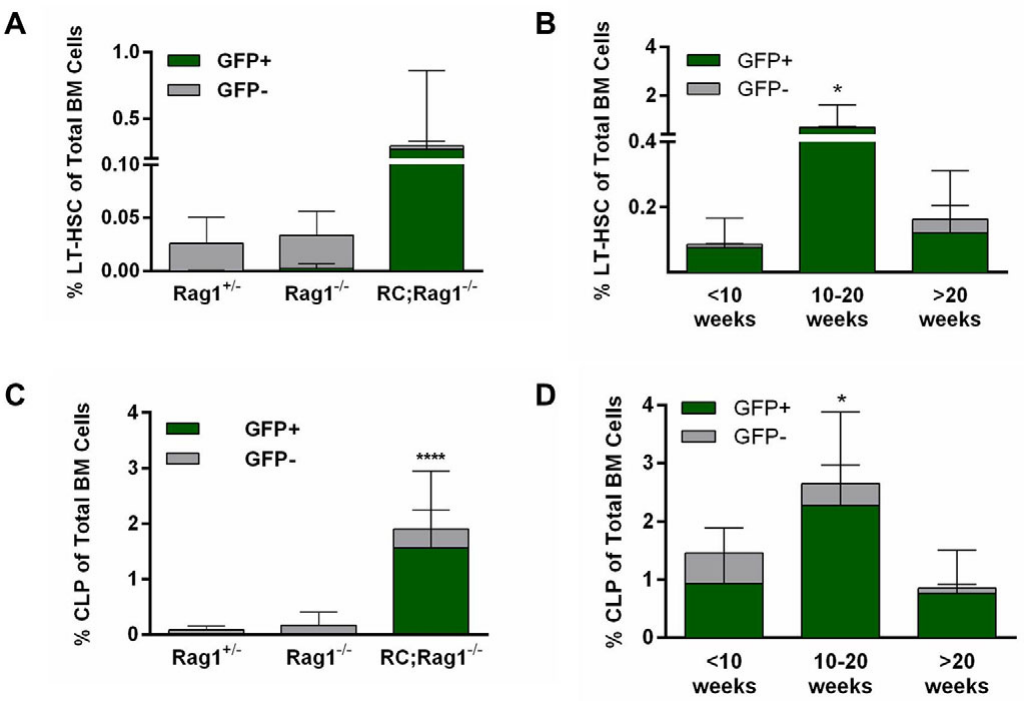
**Hematopoietic stem and progenitor cells are expanded in RAG recombination-impaired R26PR;cre mice.** (A) Percentage of long-term hematopoietic stem cells (LT-HSCs, Lin^−^c-kit^hi^Sca1^hi^ Flt3^−^CD34^−^) in whole bone marrow (BM) of *Rag1*^+/−^ (*n*=7), *Rag1*^−/−^ (*n*=18), and R26PR;MMTV-cre;*Rag1*^−/−^ (*n*=15) mice at the time of censor. (B) Percentage of LT-HSCs in BM of R26PR;MMTV-cre;*Rag1*^−/−^ mice, binned into <10 weeks (*n*=4), 10-20 weeks (*n*=5), and >20 weeks (*n*=6) age groups. (C) Percentage of common lymphoid progenitors (CLPs, Lin^−^IL7Rα^+^c-kit^+^Sca1^+^) in whole bone marrow of *Rag1*^+/−^ (*n*=7), *Rag1*^−/−^ (*n*=18), and R26PR;MMTV-cre;*Rag1*^−/−^ (*n*=15) mice at the time of censor. (D) Percentage of CLPs in bone marrow of R26PR;MMTV-cre;*Rag1*^−/−^ mice, binned into <10 weeks (*n*=4), 10-20 weeks (*n*=5), and >20 weeks (*n*=6) age groups. GFP-positive and -negative cells are displayed as green and gray bars, respectively, and mean±s.d. is shown for all graphs. RC, R26PR;MMTV-cre. *P≤0.05, ****P≤0.0001.

As R26PR;MMTV-cre;*Rag1*^−/−^ mice did not develop leukemia, we determined hematopoietic phenotypes by performing flow cytometry for stem and progenitor cells in the bone marrow. The gating strategy for determining the percentage of cells with the immunophenotype of LT-HSCs and CLPs is outlined in Figs S4 and S5, respectively. Overall, R26PR;MMTV-cre;*Rag1*^−/−^ mice appeared to have an increase in the number of LT-HSC-like cells (mean 0.3% vs 0.02-0.05%) compared to all other genotypes, and nearly all of these cells were GFP-positive, indicating that they were derived from cells with an activated R26PR allele (Fig. 4A). However, this difference was not statistically significant due to a large range, which seemed to trend with age. To distinguish between these age-related differences, R26PR;MMTV-cre;*Rag1*^−/−^ mice were binned into three groups; <10 weeks, 10-20 weeks, and >20 weeks. Young animals (<10 weeks) and older animals (>20 weeks) had significantly fewer GFP-positive LT-HSC-like cells than animals aged 10-20 weeks (mean 0.075 and 0.12% vs 0.66%, respectively; Fig. 4B).

We also evaluated the distribution of CLP-like cells among the genotypes, and found that the R26PR;MMTV-cre;*Rag1*^−/−^ group had significantly more CLP-like cells (mean 1.9% vs 0.0097-0.17%) compared to all other genotypes (Fig. 4C). The distribution of CLP-like cells within the R26PR;MMTV-cre;*Rag1*^−/−^ group was less variable than the LT-HSC-like cell distribution, but the percentage of GFP-positive CLP-like cells was still significantly lower at <10 weeks and >20 weeks of age as compared with animals aged 10-20 weeks (mean 0.93% and 0.76% vs 2.28%, Fig. 4D). The expansion of LT-HSC-like cells that contribute to the CLP population is consistent with our previously published report (Carofino et al., 2013); however, R26PR;cre mice typically succumb to T-ALL with short latency, preventing an examination of such cells over time. Therefore, this is the first time that an age-dependent reduction in LT-HSC-like cells could be noted in mice carrying R26PR;cre.

## DISCUSSION

**Fig. 5.**
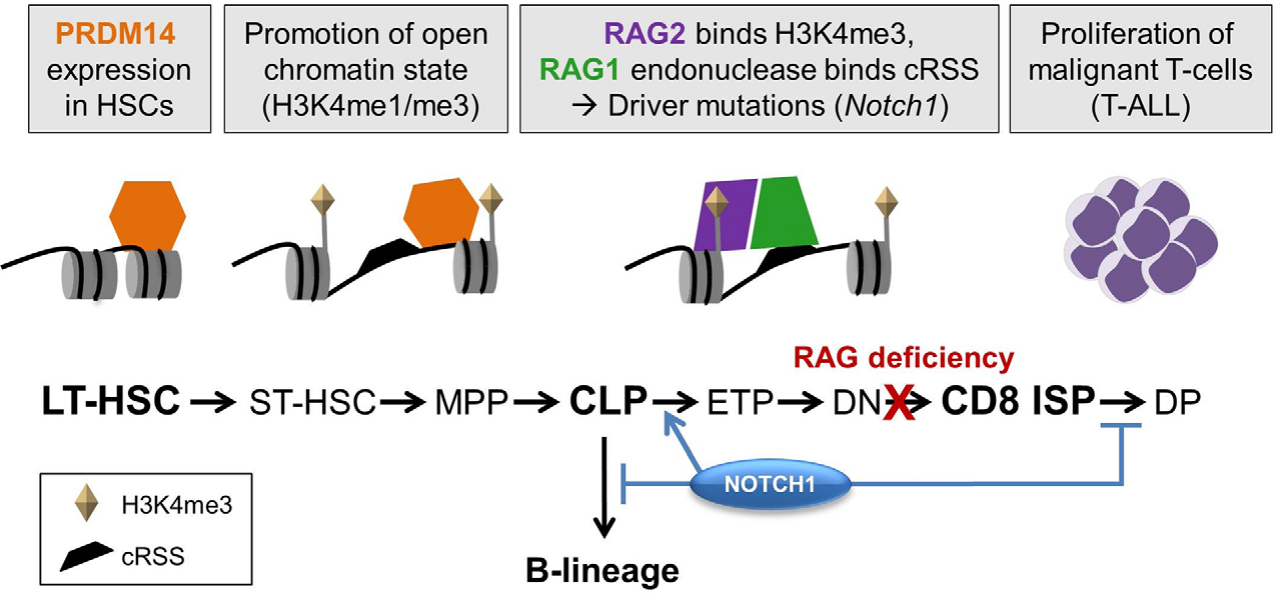
**Proposed model for PRDM14-induced leukemogenesis.** Gray boxes describe the proposed progression of events during PRDM14-induced leukemogenesis in the mouse. PRDM14 binds intron 4 of *Notch1* in HSCs, which likely allows for an open chromatin conformation, potentially by modification of H3K4, as lymphoid development proceeds. In lymphoid cells, RAG1 and RAG2 are present and can bind cRSSs to catalyze a double-stranded DNA break, leading to site-specific deletion. At *Notch1*, the deletion promotes expression of an isoform that does not require ligand, and NOTCH1 expression promotes T-lymphoid development at the expense of B-cell development. Although tumors do not develop in the absence of RAG1, T-lymphocytes arrest prior to the ISP stage of development, and *Notch1* deletions do not occur. Short-term hematopoietic stem cell (ST-HSC), multipotent progenitor (MPP), double positive T-cell (DP), immature single positive T-cell (ISP).

Here, we showed that PRDM14-induced T-ALLs were driven by activated NOTCH1 through a RAG-mediated deletion-based mechanism. Our data suggest that *Prdm14* may promote epigenetic changes that lead to the expansion of proliferating progenitor cells, which are susceptible to accumulating driver mutations such as those that occurred within *Notch1* (Fig. 5). Furthermore, PRDM14 bound to a region within the fourth intron of *Notch1* prior to leukemia onset, and may have promoted epigenetic changes that enhanced the accessibility of the *Notch1* cRSSs to the RAG complex (Ji et al., 2010). Both the *Notch1* deletion breakpoints and the downstream promoter were enriched for H3K4me3, and H3K4me1/me3 was globally enriched in PRDM14-induced T-ALLs. Whether the effect of PRDM14 on NOTCH1 activation is direct or indirect, it is clear that the overexpression of *Prdm14* dramatically alters NOTCH1-driven T-ALL latency and penetrance. Among mouse models driven by NOTCH1 through various mechanisms, only the R26PR;cre model is completely penetrant, has a median survival of less than two months, and drives aberrant *Notch1* expression from its endogenous locus (Table 1).

**Table 1.**
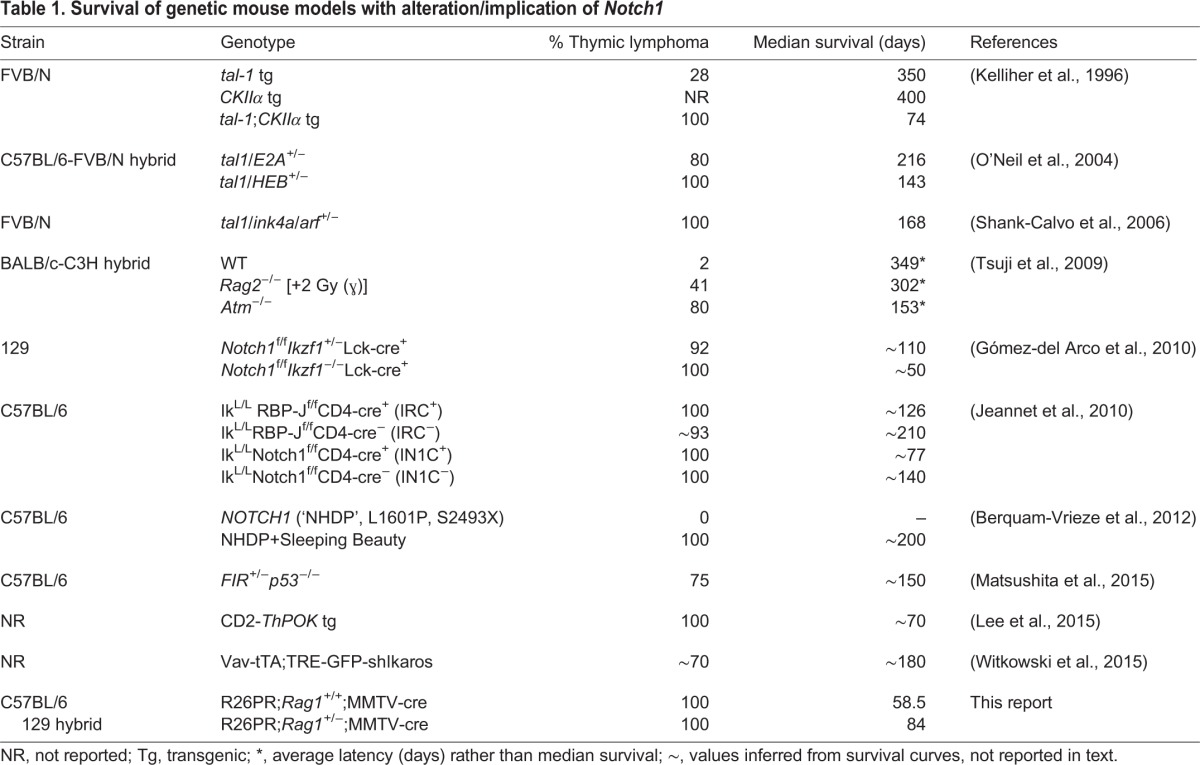
**Survival of genetic mouse models with alteration/implication of *Notch1***

The mechanism by which PRDM14 influences the epigenetic changes observed in this model remains unclear. We were unable to demonstrate HMTase activity for PRDM14 *in vitro*, but this may have resulted from the addition of tags to the recombinant protein or lack of necessary co-factors. Although PRDM proteins belong to the same family as SET domain-containing HMTases, a predominant lack of HMTase activity among PRDM proteins has lead to speculation that PRDM proteins may function by methylating non-histone targets or associating with other enzymatically active proteins (Fog et al., 2012). Indeed, PRDM14 has been shown to mediate gene repression through association with PRC2 (Chan et al., 2013). Recently, PRDM14 has also been shown to interact with MTGR1, which seems to promote gene repression through the recruitment of histone deacetylases (HDACs) to bound targets (Nady et al., 2015). Nady et al. also failed to detect PRDM14 HMTase activity *in vitro*; further, no HMTase interactions were found in ESCs. Together, these data suggest that the PRDM14 pre-SET domain structure is not compatible with S-adenosyl-L-methionine (SAM) binding, which is necessary for methylation. Although PRDM14 has largely been described as a factor that maintains stemness through the repression of the differentiation program, it has also been shown to promote the activation of ESC maintenance genes (Ma et al., 2011), but the precise mechanism of this activity has not been described. We suggest that the activity of PRDM14 is largely context-specific, so a unique set of PRDM14 binding partners and activities may be present in pre-leukemic and tumor cells, and may include HMTases.

Breeding the R26PR;MMTV-cre mouse line into a *Rag1*-deficient background was sufficient to prevent NOTCH1-driven T-ALL, both because RAG-mediated *Notch1* deletions did not occur and because thymocyte development was blocked at DN3/4 due to failed TCR expression. However, the expansion of LT-HSC-like and CLP-like cells that results from *Prdm14* overexpression in the bone marrow occurred independently of *Rag1* status, but may have resulted from PRDM14-mediated transcriptional activation of *Notch1* in HSCs. Stier et al. reported an identical increase in HSCs and preferential lymphoid over myeloid differentiation upon transducing *Rag1^−/−^* stem cell-enriched BM with retroviral vectors expressing constitutively active NOTCH1 (Stier et al., 2002). In this transduction model, NOTCH1, which is known to inhibit hematopoietic progenitor differentiation, increases HSC numbers by promoting HSC self-renewal.

Other PRDM proteins are important for HSC self-renewal, maintenance, and function (Pinheiro et al., 2012). *Prdm3* deficiency leads to a complete loss of long-term repopulation capacity, suggesting that the normal role of PRDM3 is to maintain LT-HSC quiescence (Zhang et al., 2011). Loss of *Prdm16* also leads to a reduction in HSC numbers and a reduction in long-term repopulation capacity (Aguilo et al., 2011). In addition to the increase in LT-HSC-like cells in R26PR;cre;*Rag1*^−/−^ mice, we noted that younger mice (<10 weeks) and older mice (>20 weeks) had significantly fewer LT-HSC-like cells than animals aged 10-20 weeks; thus, the peak of LT-HSC-like cell expansion occurred between 10 and 20 weeks. This observation suggests that overexpression of PRDM14 may promote HSC proliferation at the expense of self-renewal. HSC exhaustion secondary to stem and progenitor cell expansion is a common feature of leukemia development, perhaps resulting from disrupted HSC/niche interactions, but is often overcome by cooperating mutations that restore self-renewal during cancer development (Jacob and Osato, 2009). Furthermore, other SET domain-containing proteins, such as MLL, are critical for regulating HSC self-renewal (McMahon et al., 2007), reinforcing the notion that PRDM14 may also play a normal physiological role in HSCs or usurp the function of other SET domain-containing proteins when aberrantly expressed. Mice overexpressing PRDM14 in HSCs typically succumb to leukemia by 8-weeks of age, precluding our ability to make this observation previously and making the *Rag1^−/−^* genetic background ideal for future studies to fully dissect the function of PRDM14 in hematopoietic progenitor cells.

The rapid, fully penetrant onset of T-ALL in the R26PR;cre model may result from the ability of PRDM14 to couple stem cell expansion with genomic instability. Expression of *Prdm14* in lymphoid progenitors causes widespread DNA damage as evidenced by array comparative genomic hybridization (aCGH) analysis of tumors (Simko et al., 2012). In pluripotent cells, histone marks associated with PRDM14 are predicted to poise genes for transcriptional activity, with H3K4me1 and H3K4me3 predicting an open chromatin state. Recombination at cRSSs can lead to extensive genomic damage in lymphocytes if the cRSSs are present at transcriptionally active sites, since RAG2 binds to H3K4me3 (Teng et al., 2015). *Prdm14* misexpression could also promote RAG-dependent driver mutations at loci other than *Notch1* in lymphoid cells to cause the other lymphoid-lineage leukemias we previously observed in other PRDM14-induced models (Dettman and Justice, 2008; Dettman et al., 2011). Our previous aCGH data of PRDM14-induced pre-B and pre-T ALLs found a large number of common deletions and amplifications in pre-B-cell and mixed lineage cell tumors, yet pre-T-cell tumors were relatively free of common copy number variants with the exception of those at *Notch1*. Common genomic rearrangements in pre-B-cell and mixed lineage tumors occurred at sites bound by PRDM14, many of which contain cRSSs, including cyclin dependent kinase 2a (*Cdkn2a*), a common driver in cancers (Simko et al., 2012). Therefore, in lymphoid cells, PRDM14 can promote driver mutations by hijacking normal DNA recombination machinery. These data reveal a potential mechanism for the promotion of DNA damage in lymphocytes through the misregulation of epigenetic regulators.

Our ultimate goal was to understand how PRDM14 initiates cancer and to exploit this knowledge for cancer treatment. Gamma-secretase inhibitors, which inhibit NOTCH1 cleavage and activation, have had limited clinical success, primarily due to dose-limiting gastrointestinal toxicities. Because the R26PR;cre model develops fully penetrant, rapid-onset T-ALL that is driven by NOTCH1, it is ideal for the preclinical testing of novel drugs. Although RAG-mediated *NOTCH1* deletions have not been reported in human T-ALL, the resultant accumulation of ligand-independent NOTCH1 protein also occurs as a consequence of the most common human *NOTCH1* mutations (Weng et al., 2003; Malecki et al., 2006). Therefore, this model can be used to test the efficacy of novel gamma secretase inhibitors or NOTCH1 inhibitors. Furthermore, the inducible FLAG-PRDM14 mouse model will allow us to perform a direct assessment of genome-wide PRDM14 binding during tumorigenesis. This will allow us to fully dissect the molecular mechanisms of PRDM14-mediated tumor initiation, progression, and relapse in leukemia, as well as in solid tumor models.

## MATERIALS AND METHODS

### Mice

All mouse experiments were carried out at Baylor College of Medicine (BCM) and The Hospital for Sick Children with the approval of their respective Institutional Animal Care and Use Committees. All mice were housed in a pathogen-free facility. Mouse lines included: *Gt(ROSA)26Sor^tm1Jus^* – ‘R26PR’ (Carofino et al., 2013), *Gt(ROSA)26Sor^tm2Jus^* – ‘R26FLPR’ (both generated by Justice Laboratory), Tg(MMTV-cre)4Mam/J – ‘MMTV-cre’ (The Jackson Laboratory, Bar Harbor, Maine, US), Tg(Mx1-cre)1Cgn/J – ‘Mx1-cre’ (from Dr Margaret A. Goodell, Baylor College of Medicine, Houston, Texas, US), and *Rag1^tm1Mom^*/J – ‘*Rag1*^−/−^’ (from Dr David Corry, Baylor College of Medicine, Houston, Texas, US). All lines were on a C57BL/6 (J or N) background, with the exception of MMTV-cre (B6.129 F1 hybrid). The R26FLPR line was generated as previously described (Carofino et al., 2013), except for the addition of an N-terminal FLAG tag and the addition of P2A-eGFP rather than IRES-eGFP. P2A-eGFP was amplified from OCT4-2A-eGFP-PGK-Puro, which was a gift from Rudolf Jaenisch (Addgene plasmid # 31938). Mx1-cre was activated in both the R26PR;Mx1-cre and R26FLPR;Mx1-cre lines via intraperitoneal injection of 250 µg polyinosinic:polycytidylic acid (pIpC) (Sigma-Aldrich), with three doses spaced two days apart and one dose, respectively, at 8 weeks of age.

### Polymerase chain reaction (PCR)

Genotyping PCR was performed using Apex Hot Start Master Mix (Genesee Scientific). Primer sequences for detecting the Cre-deleted R26PR and R26FLPR alleles were previously described (Carofino et al., 2013). *Notch1* deletions were detected as previously described (Ashworth et al., 2010).

### Chromatin immunoprecipitation

Chromatin immunoprecipitation (ChIP) on thymi was performed using the MAGnify Chromatin Immunoprecipitation System (Life Technologies) according to the manufacturer's instructions. ChIP on bone marrow was performed using standard protocols. Briefly, cells were fixed at room temperature for 10 min with 1% formaldehyde. Cells were lysed and DNA was sheared by sonication. Lysates were incubated overnight with antibody and subsequently reverse cross-linked for 18 h at 65°C. After RNaseA and proteinase K treatments, immunoprecipitated DNA was isolated and qPCR was performed with data analyses conducted on an Applied Biosystems Viia7. Antibodies were from Abcam: H3 (ab1791), H3K4me3 (ab8580), Cell Signaling Technology: IKAROS (9034), and Sigma-Aldrich: FLAG M2 (F1804).

### Quantitative RT-PCR

Total RNA was extracted from frozen thymi using TRIzol Reagent (Life Technologies), and was reverse transcribed using the SuperScript III First-Strand Synthesis System (Life Technologies). Immunoprecipitated DNA or cDNA was amplified using Power SYBR Green PCR Master Mix (Life Technologies) and gene-specific primers (available upon request). Ratiometric qRT-PCR for *Notch1* was performed as previously described (Ashworth et al., 2010). Amplification and data analyses were conducted on a Rotor-Gene Q (Qiagen). Relative gene expression was calculated by the ΔΔC_T_ method.

### Flow cytometry

Flow cytometry was performed on total bone marrow or thymic single cell suspensions. Cells were stained with fluorophore-conjugated antibodies from eBioscience: CD4-Alexa Fluor 700 (56-0041), CD4-PE (12-0041), CD8-eFluor 450 (48-0081), CD24-PE (12-0241), CD25-APC (17-0251), CD34-Alexa Fluor 700 (56-0341), CD44-PE-Cy7 (25-0441), c-Kit-APC-eFluor 710 (47-1171), Flt3-PE (12-1351), IL-7Rα-eFluor 450 (48-1271), Sca1-APC (17-5981) or BD Pharmingen: Lineage Cocktail-APC (558074), Lineage Cocktail-PerCP-Cy5.5 (561317), and TCRβ-APC (561080). Samples were analyzed on an LSR Fortessa flow cytometer (BD Biosciences) in the Flow Cytometry and Cell Sorting Core at BCM. Data interpretation used FACSDiva (BD Biosciences) and FlowJo (TreeStar Inc.) software.

### Western blot

Protein was prepared from frozen thymi using T-PER (Thermo Scientific) with protease inhibitors (Roche). Histones were purified from whole cell lysates using the Histone Purification Mini Kit (Active Motif). Antibodies were used at manufacturer-recommended concentrations from Active Motif: H3K4me1 (39297), H3K4me3 (39915), H3K9me2 (39753), H3K27me3 (39155), H3 (61277), Cell Signaling Technology: IKAROS (5443), Cleaved NOTCH1 (Val1744) (4147), Sigma-Aldrich: FLAG M2 (F1804), Bio-Rad: HRP-goat-anti-mouse, and Jackson Immunoresearch: HRP-goat-anti-rabbit.

### Recombinant protein production and purification

*Prdm14* was PCR amplified and cloned into the 6×His-GST-tag-containing baculovirus transfer vector pAcGHLT-B (BD Biosciences) using In-Fusion cloning (Clontech). Baculovirus clones were used to infect Sf9 insect cells by the Monoclonal Antibody/Protein Expression Core at BCM. PRDM14 or G2T-GST (GST-only control) baculovirus was used to infect Sf9 cells at a molarity of infection (MOI) of 1 for 48 or 72 h of culture in 500 ml spinner vessels. 6×His-GST-PRDM14 was purified from cell pellets using the BD GST Purification Kit (BD Biosciences) and desalted/concentrated using Amicon Ultra-4 10,000 NMWL filter devices (Millipore). Glycerol was added to a final concentration of 10% and protein lysates were stored at −80°C until use.

### Histone methyltransferase assay

Histone methyltransferase assays were performed as previously described (Fingerman et al., 2008), except that 10× HMTase buffer (NEB) and 6×His-GST-PRDM14 or GST protein derived from baculovirus-infected Sf9 cells was utilized instead of *E. coli*-derived protein lysates.

### Data analysis

Graphical and statistical analyses were performed with Prism (GraphPad). The log-rank test was performed for survival analysis, and a one-way ANOVA with Dunnett's correction for multiple comparisons was performed on data with three or more groups. All other analyses with two groups used the Student's *t*-test.
